# Holistic analysis of lysine acetylation in aquaculture pathogenic bacteria *Vibrio alginolyticus* under bile salt stress

**DOI:** 10.3389/fvets.2023.1099255

**Published:** 2023-04-27

**Authors:** Xing Xiao, Wanxin Li, Yanfang Pan, Junlin Wang, Zhiqing Wei, Shi Wang, Na Wang, Jichang Jian, Huanying Pang

**Affiliations:** ^1^Fisheries College of Guangdong Ocean University & Southern Marine Science and Engineering Guangdong Laboratory (Zhanjiang), Zhanjiang, China; ^2^Guangdong Provincial Key Laboratory of Aquatic Animal Disease Control and Healthy Culture, Key Laboratory of Control for Diseases of Aquatic Economic Animals of Guangdong Higher Education Institutes, Zhanjiang, China; ^3^School of Public Health, Fujian Medical University, Fujian, China; ^4^Chinese Academy of Inspection and Quarantine, Beijing, China

**Keywords:** *Vibrio alginolyticus*, bile salt, acetylome, post-translational modification, virulence

## Abstract

Lysine acetylation modification is a dynamic and reversible post-translational modification, which plays an important role in the metabolism and pathogenicity of pathogenic bacteria. *Vibrio alginolyticus* is a common pathogenic bacterium in aquaculture, and bile salt can trigger the expression of bacterial virulence. However, little is known about the function of lysine acetylation in *V. alginolyticus* under bile salt stress. In this study, 1,315 acetylated peptides on 689 proteins were identified in *V. alginolyticus* under bile salt stress by acetyl-lysine antibody enrichment and high-resolution mass spectrometry. Bioinformatics analysis found that the peptides motif ^****^A^*^Kac^****^ and ^*******^Kac^****^A^*^ were highly conserved, and protein lysine acetylation was involved in regulating various cellular biological processes and maintaining the normal life activities of bacteria, such as ribosome, aminoacyl-tRNA biosynthesis, fatty acid metabolism, two-component system, and bacterial secretion system. Further, 22 acetylated proteins were also found to be related to the virulence of *V. alginolyticus* under bile salt stress through secretion system, chemotaxis and motility, and adherence. Finally, comparing un-treated and treated with bile salt stress lysine acetylated proteins, it was found that there were 240 overlapping proteins, and found amino sugar and nucleotide sugar metabolism, beta-Lactam resistance, fatty acid degradation, carbon metabolism, and microbial metabolism in diverse environments pathways were significantly enriched in bile salt stress alone. In conclusion, this study is a holistic analysis of lysine acetylation in *V. alginolyticus* under bile salt stress, especially many virulence factors have also acetylated.

## Introduction

With the development of biotechnology development and related research, more than 200 types of post-translational modifications (PTMs), such as phosphorylation, methylation, acetylation, succinylation, and glycosylation, and a large number of PTM sites have been identified (1, 2). Acetylation is an evolutionarily conserved, abundant, and ubiquitous protein post-translational modification that plays a crucial role in the regulation of bacterial enzymatic activity, cellular physiology, metabolic processes, and virulence (3–5). Before 2008, only a few bacterial acetylated proteins had been identified and characterized, then with the rapid development of high-affinity immunoisolation using antibodies to enrich acetylated proteins/peptides from complex samples and HPLC-MS/MS technology, many research groups conducted a global screening and characterization of lysine acetylation in bacteria, such as *Escherichia coli, Bacillus subtilis*, and *Mycobacterium tuberculosis* (6–8), as well as aquatic pathogenic bacteria *Aeromonas hydrophila, Vibrio mimicus, Vibrio parahaemolyticus, and Vibrio alginolyticus* (9–12), etc., to provide a framework for the systematic analysis of the physiological role and regulatory mechanism of lysine acetylation in bacteria. And as the number of identified acetylation sites and proteins increases, important information such as protein interaction network, localization and gene ontology annotation can be analyzed more comprehensively (2). However, how protein acetylation in bacteria affects global cellular metabolism as well as its complex regulatory mechanisms and physiological effects are still unclear and required further research.

*Vibrio alginolyticus* is a halophilic Gram-negative bacterium, is widely distributed in the sea, inshore and river mouth areas. It is not only the main pathogen of mariculture economic animals, such as oysters, groupers and *Penaeus vannamei* (13–15), causing huge economic losses to the aquaculture industry and posing a great threat to the breeding environment, but also the key reason for acute gastroenteritis, diarrhea, and even septicemia in humans caused by exposure to contaminated seafood all over the world (16, 17). It mainly produces pathogenic effects by producing pathogenic factors that damage host somatic cells and interfere with metabolism. In *V. alginolyticus* common virulence factors genes including virulence expression regulatory genes (*toxR*), outer membrane protein (*ompW, ompR, ompN*), adhesion (*flrA, flrB, flrC*), hemolysin (*thd*), secretion system (*tyeA, Val1686*, Val1680), and so on (2, 18–22).

Bile salt is the main component of bile, which can affect cell membrane protein and phospholipid structure, thereby disrupting cell membranes, damaging nucleic acids, causing redox stress, and triggering the expression of bacterial virulence *in vivo* (23, 24). Many gut bacteria adapt to bile salt stress by decreasing membrane permeability, stimulating efflux pumps, inducing biofilm formation, and up regulating redox and DNA damage repair genes (23), such as the production of c-di-AMP in *Clostridium difficile* makes cells sensitive to osmotic stress, and then regulates bile salt stress (25), *E. coli* is protecting itself by the Rob-mediated upregulation of AcrAB against the harmful effects of bile salts in the intestinal tract (26). In *V. parahaemolyticus*, genes *vtrC, vtrA* and *vtrB* are necessary to activate type III virulence secretion system 2 in response to bile salts (27). However, the mechanism of pathogenic bacteria sensing bile salts, whether directly by binding to signaling proteins or indirectly by sensing cell damage, requires further in-depth study.

More and more studies have found that protein acetylation may be related to bacterial virulence, especially the acetylation of bacterial virulence factors. Such as acetylation of GtfB and GtfC will change the formation of biofilm of *S. mutans*, thus regulating its virulence and pathogenicity (28), acetylation can regulate the activity of PhoP, impairs its DNA-binding ability, and resulting in attenuated virulence of *S. typhimurium* (29), the reversible acetylation mediated by CobB and YfiQ can regulate the *psaABCDEF* operon, thus affecting the expression of Psa fimbriae protein, and ultimately affecting *Y. pestis* virulence (30). Acetylation acts as a fine-tuning switch that can regulate the virulence of bacteria.

Therefore, in this study, we treated *V. alginolyticus* with bile salt to extract the whole protein of bacteria, and identified 1,315 acetylated peptides and 689 acetylated proteins by acetyl-lysine antibody enrichment and LC-MS/MS analysis technology. The acetylated peptide analysis found that the sequence motifs ^****^A^*^Kac^****^ and ^*******^Kac^****^A^*^ were obviously conserved, while the acetylated proteins were mainly involved in ribosome, two-component system, aminoacyl tRNA biosynthesis, fatty acid metabolism, and bacterial secretion system processes. Through screening with VFDB database, 22 acetylated proteins were found to be related to virulence. The lysine acetylation overlapping proteins were further compared and analyzed under bile salt stress and unstressed environments. Bioinformatics analysis found that most of the overlapping acetylated proteins were involved in the regulation of ribosomes, antibiotic biosynthesis, pyrimidine metabolism, and purine metabolism. In conclusion, this study provides a theoretical basis for future research on the regulatory mechanism of bile salts in *V. alginolyticus*, especially in the study of bacterial physiological functions in terms of protein post-translational modification.

## Materials and methods

### Bacterial strains and total protein extraction

*Vibrio alginolyticus* HY9901 was isolated from *Lutjanus erythopterus*, and stored in our laboratory (31). Its optimum culture temperature is 28°C. Pick monoclonal colonies from TSB (Tryptone Soya Broth, Oxoid U.S.A., Inc., Columbia, Md) medium plates into TSB liquid medium for overnight culture. The next day, overnight culture transfer by 1% to a fresh TSB medium containing 0.04% bile salts (Sigma-Aldrich, St. Louis, MO) (32) and culture until the OD was about 1.0, and then collect cells were by centrifugation. The pellet was washed twice with pre-cooled PBS (Phosphate Buffered Saline) and dissolved in lysis buffer [containing 100 mM NH_4_HCO_3_ (pH 8), 6 M Urea and 0.2% SDS], followed by 5 min of ultrasonication on ice. The lysate was centrifuged at 12,000 g for 30 min at 4°C and collect the supernatant as the whole protein sample. The protein was reduced with 2 mM DTT (Thermo Fisher, Waltham, Massachusetts, USA) for 1 h at 56°C, and subsequently alkylated with sufficient Iodoacetamide (IAA, Thermo Fisher, Waltham, Massachusetts, USA) for 1 h at room temperature in the dark. Then 4 times volume of pre-cooled acetone was mixed with samples by well vortex and incubated at −20°C for at least 2 h. Sample was then centrifuged and the precipitation was collected. After washing twice with pre-cold acetone, the pellet was dissolved by dissolution buffer [6 M urea in 0.1 M triethylammonium bicarbonate (TEAB, pH 8.5), Thermo Fisher, Waltham, Massachusetts, USA]. Protein concentration was determined by Bradford protein assay.

### Peptide preparation

Ten milligrams of protein was digested with Trypsin Gold (Promega, Madison, Wisconsin, USA) at 1:50 enzyme-to-substrate ratio in 50 mM TEAB buffer. After 16 h of digestion at 37°C, an equal volume of 1% formic acid solution was added and centrifuged at room temperature for 5 min at 12,000 g. Then the supernatant was desalted using a Sep-Pak Vac C18 cartridge (Thermo Fisher, Waltham, Massachusetts, USA), and washed three times with a cleaning solution containing 0.1% formic acid and 4% acetonitrile to remove the high urea. Finally, an eluent buffer containing 0.1% formic acid and 75% acetonitrile was added to elute the desalted peptide, and dried by vacuum centrifugation (Labogene, Denmark).

### Immunoaffinity enrichment of lysine-acetylated peptides

Lyophilized peptide segments were dissolved with MOPS IAP solution (Tris-HCl, pH 7.0) containing 50 mM MOPS, 10 mM KH_2_PO_4_ and 50 mM NaCl, and centrifuged at 4°C and 12,000 g for 5 min. The supernatant was mixed with Acetyl-Lysine Motif [Ac-K] Immunoaffinity Beads (CST #13416, Cell Signaling Technology, Danvers, Massachusetts, USA) and washed three times with 1 × PBS, followed by incubation at 4°C for 2.5 h. After incubation, the sample was centrifuged at 4°C and 3,000 g for 30 s, then the supernatant was discarded and beads were washed in precooled MOPS IAP buffer, then in H_2_O for 3 times, respectively. Finally, the peptides were eluted with 0.15% TFA. After mixing and incubation at room temperature for 10 min, centrifuged at 3,000 g for 30 s, and the elution was repeated 3 times. The eluted samples were pooled and centrifuged at 5,000 g for 5 min, and the supernatant were desalted using peptide desalting spin columns (Thermo Fisher, Waltham, Massachusetts, USA) and lyophilization.

### LC-MS/MS analysis

Use 10 μl of 0.1% FA (formic acid) buffer to resuspend the sample, centrifuge at 10,000 rpm for 5 min at room temperature, and take 1 μg of supernatant sample for proteomics analyses using an EASY-nLCTM 1200 UHPLC (ultra-high pressure liquid chromatography) system (Thermo Fisher, Waltham, Massachusetts, USA) coupled with an Orbitrap Q Exactive HF-X mass spectrometer (Thermo Fisher, Waltham, Massachusetts, USA) operating in the DDA (data-dependent acquisition) mode. Refer to the previous description for specific details (12).

### Data preprocessing

The database used is the Uniprot_*Vibrio alginolyticus* protein database (4,338 sequences). The raw files were directly imported into Proteome Discoverer 2.2 software for database search, peptide and protein quantification. Filtered the search results including the peptide spectrum matches (PSMs) with a reliability of more than 99%, the proteins containing at least one unique peptide, and to remove the peptides and proteins with an FDR (False discovery rate) >1%. After the database search, a series of quality control needs to be carried out, including peptide length distribution, precursor ion mass tolerance distribution, Unique peptide number distribution, protein coverage distribution, and protein molecular weight distribution. The mass spectrometry proteomics data have been deposited to the ProteomeXchange Consortium (http://proteomecentral.proteomexchange.org) via the iProX partner repository (33) with the dataset identifier PXD035599.

### Bioinformatics analysis

Sequence motifs associated with protein acetylation modification were analyzed using online software MoMo (Version 5.4.1, https://meme-suite.org/meme/index.html). Analysis of cellular components (CC), molecular functions (MF), biological processes (BP) and Kyoto Encyclopedia of Genes and Genomes (KEGG) were performed using the online software Omicsbean (http://www.omicsbean.cn/). The annotation of subcellular localization was performed using the Cell-mPLOC 2.0 (34). The COG database of NCBI (https://www.ncbi.nlm.nih.gov/COG/) was used to analyze the Cluster of Orthologous Groups of proteins (COG). STRING (https://cn.string-db.org/) combined with Cytoscape_3.1.7 software were used for protein-protein interaction analysis and graphical visualization. The Virulence Factor Database (VFDB) database (http://www.mgc.ac.cn/VFs/main.htm) was used to screen virulence factors. Use GraphPad Prism 8 software to generate the above analysis results.

### Co-Immunoprecipitation and western blotting

The whole protein (500 μg) of *V. alginolyticus* under bile salt stress was incubated with specific polyclonal antibodies OmpN, OmpR, and GrpE at 4°C overnight to precipitate target proteins, as previously described (10). Briefly, the protein A/G beads were washed three times with pre-cooled PBS buffer (137 mM NaCl, 2.7 mM KCl, 4.3 mM Na_2_HPO_4_, 1.4 mM KH_2_PO_4_, pH 7.2–7.4) and then added to the lysate for incubation at 4°C for 2 h. The beads were centrifuged at 4°C and 2,500 g for 5 min to remove the supernatant, and washed five times with pre-cooled PBS buffer. In total, 50 μl loading buffer (containing 250 mM Tric-HCl pH 6.8, 10% SDS, 0.5% bromophenol blue, 50% glycerol and adding 5% β-mercaptoethanol before use) was added and boiled for 5 min, and then run SDS-PAGE and Western blot verification. After SDS-PAGE, a Trans-Blot Turbo Transfer System (Bio-Rad, Hercules, CA, USA) was used to transfer proteins to PVDF membrane (polyvinylidene fluoride, Millipore, Billerica, MA, USA). The membrane was blocked with QuickBlock^TM^ blocking buffer (Beyotime Biotechnology Co., Ltd, Shanghai) 15 min at room temperature and probed with OmpN, OmpR, GrpE serum antibody and anti-acetyllysine mouse mAb (PTM-101, 1:5,000 diluted in QuickBlock^TM^ blocking buffer, PTM Biolabs Inc., Hangzhou, China), then incubated at 4°C for overnight The second antibody was HRP-labeled Goat Anti Rabbit IgG or Anti Mouse IgG (1:8,000 diluted in QuickBlock^TM^ secondary antibody dilution buffer) which is incubated at room temperature for 2 h, and then the membrane is washed four times with TBST (20 mM Tris, 137 mM NaCl, 0.1% Tween-20, pH 7.6). Finally, the membrane colored by claritytm Western ECL substrate (#1705061, Bio-rad Co., Ltd.). The automatic chemiluminescence image analysis system (TAN 5200, Tanon Science & Technology Co., Ltd, Shanghai, China) was used to take photos and record the experimental results.

## Results

### Identification of lysine acetylated peptides and proteins under bile salt stress in *V. alginolyticus*

**Figure 1:**
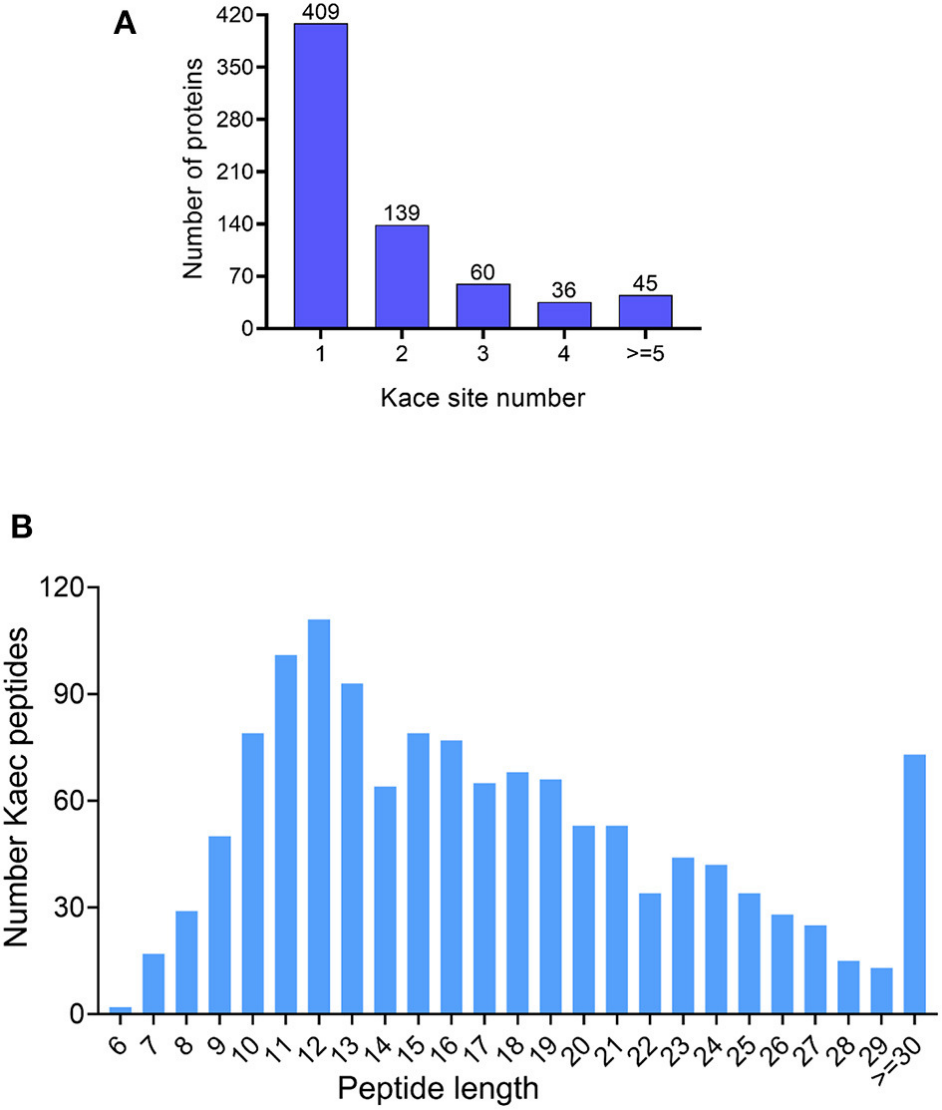
Outline of acetylome under bile salt stress in *V. alginolyticus*. **(A)** Site coverage of acetylated proteins, and **(B)** Distribution of acetylated peptide lengths.

In the study, immunoaffinity enrichment of lysine-acetylated peptides with Acetyl-Lysine Motif [Ac-K] Immunoaffinity Beads and LC-MS/MS analysis to profile the acetylated proteins and peptides under bile salt stress in *V. alginolyticus*. The analysis found that peptides and proteins with FDR < 1% had a total of 1,315 peptides and 689 proteins were acetylated under bile salt stress in *V. alginolyticus* (Supplementary Tables 1, S2). The modification sites in acetylated proteins are mainly between 1 and 12. The results showed that 409 proteins (59.36%) have only one modification site, 129 proteins (20.17%) have two sites, 60 proteins (8.71%) have three sites, 36 proteins (5.23%) have four sites, and 45 proteins (6.53%) have more than four modification sites, and the proteins RpoB and N646_ 0059 contain 12 modification sites (Figure 1A). Most of the acetylated-modified peptides are between 7 and 29 amino acids (94.3%), only two peptides are six amino acids, and 73 peptides (5.55%) are ≥30 amino acids (Figure 1B).

### Sequence motifs of lysine acetylated-peptides under bile salt stress in *V. alginolyticus*

**Figure 2:**
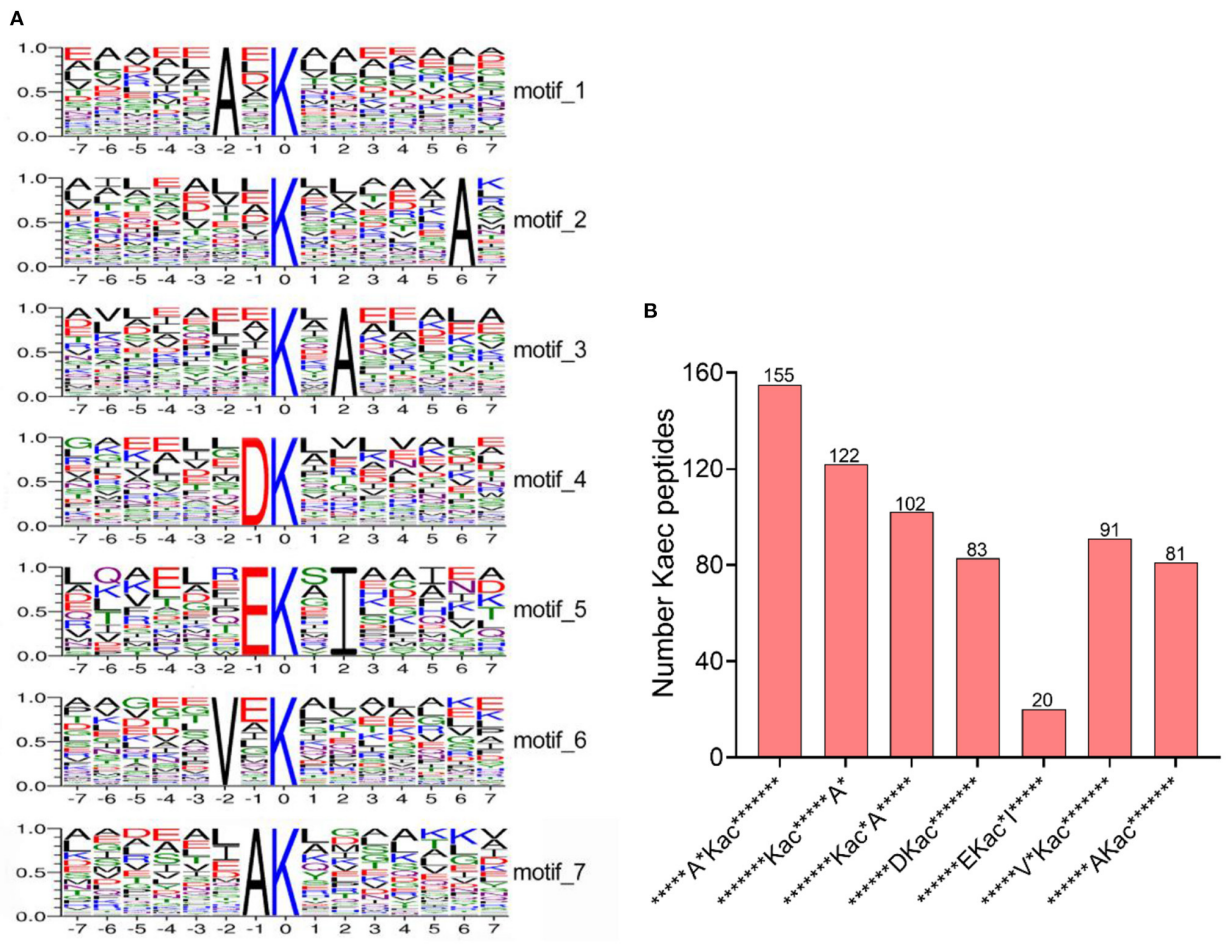
Analysis of motifs of lysine acetylated-peptides under bile salt stress in *V. alginolyticus*. **(A)** The sequence logos of the acetylation motifs identified by MoMo software. These motifs consist of 14 residues (seven amino acids upstream and seven amino acids downstream) surrounding the acetylated lysine, and **(B)** Number of acetylated peptides contained in the identified motif.

In order to identify potential motifs in acetylated peptides under bile salt stress in *V. alginolyticus*, sequence motifs (amino acid sequences from the −7 to +7 positions of the identified Kac, Kac represents lysine acetylation modification site) were extracted by online software MoMo. From 1,315 unique lysine-acetylated peptides, seven sequence motif were enriched, namely ^*****^A^*^Kac^*******^, ^*******^Kac^*****^A^*^, ^*******^Kac^*^A^*****^, ^******^DKac^*******^, ^******^EKac^*^I^*****^, ^*****^V^*^Kac^*******^ and ^******^AKac^*******^ (^*^ represents a random amino acid) (Figure 2A). Particularly, sequence motifs ^*****^A^*^Kac^*******^ (155 Kac peptides) and ^*******^Kac^*****^A^*^ (122 Kac peptides) were evidently conserved (Figure 2B). Lysine acetylation occurs preferentially in regions rich in alanine (A), such as on position −2, +6, +2, and −1. Acidic amino acid aspartate (D) and glutamate (E) residue were preferentially occupied the position −1, and valine (V) residue preferred to be on position −2 (Figure 2).

### GO and KEGG annotation and enrichment of lysine acetylated proteins under bile salt stress in *V. alginolyticus*

**Figure 3:**
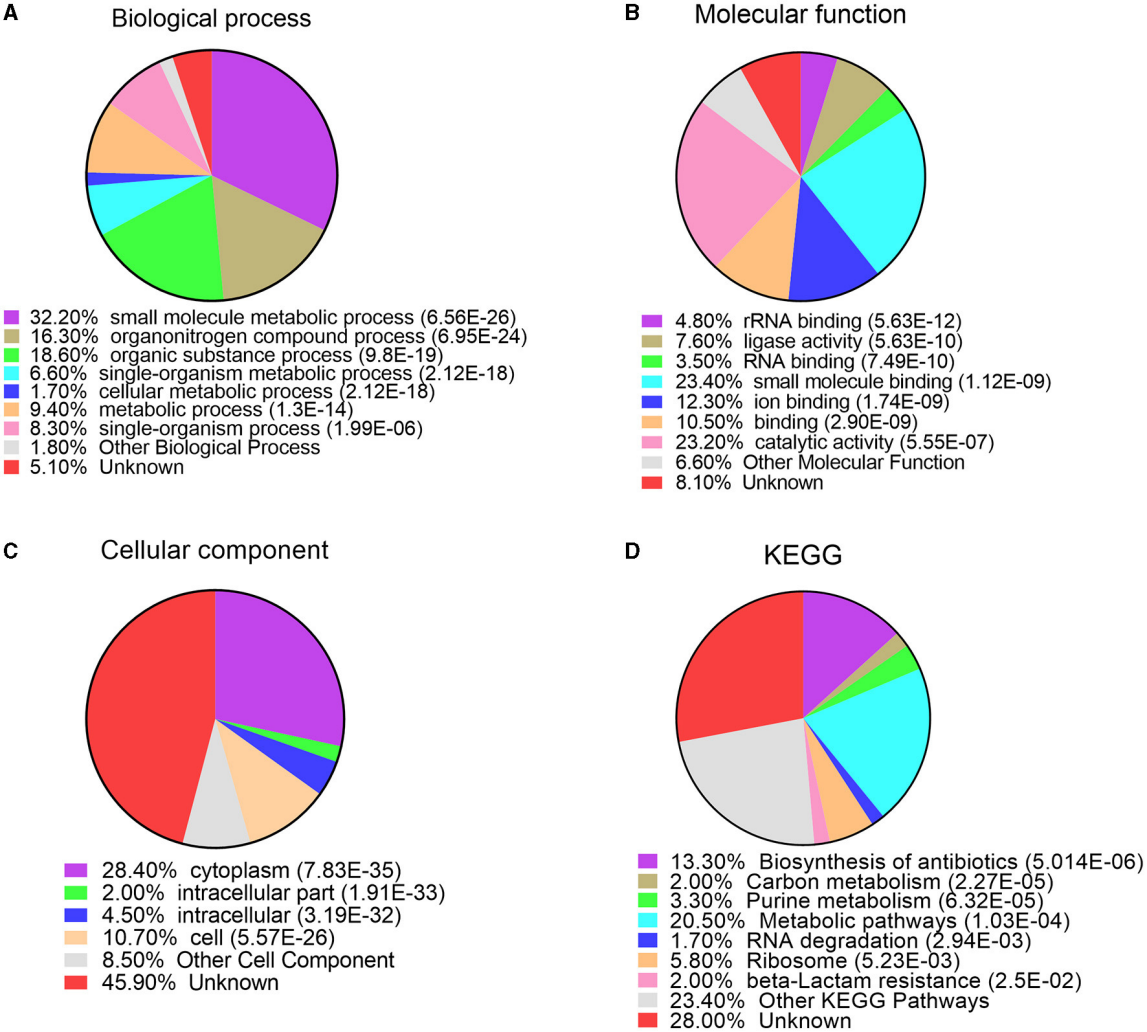
GO and KEGG enrichment analysis of lysine acetylated proteins under bile salt stress in *V. alginolyticus*. **(A)** biological process, **(B)** molecular function, **(C)** cellular component, and **(D)** KEGG pathway.

In order to better understand the function of acetylated modified proteins under bile salt stress in *V. alginolyticus*, we performed annotation and enrichment of GO (Gene Ontology) and KEGG functions of modified proteins. Go annotation includes three parts: biological processes, molecular functions and cell components. For biological processes, acetylated proteins are mainly involved in the regulation of small molecule metabolic process, organonitrogen compound metabolic process, organic substance metabolic process, single-organism metabolic process, cellular metabolic process, metabolic process, and single-organism process (Figure 3A). rRNA binding, ligase activity, RNA binding, small molecule binding, ion binding, binding, and catalytic activity were the largest enriched molecular functions (Figure 3B). For cellular components, the majority of acetylated proteins were found to enriched in cytoplasm, cell, intracellular, intracellular part (Figure 3C). Analysis results indicate that most lysine acetylated proteins are categorized as involved in small molecule metabolic processes, binding functions, and location of the cytoplasm.

In order to further insights into the functional correlation of acetylation in *V. alginolyticus* under bile salt stress, we performed KEGG pathways enrichment analysis for all identified acetylated proteins. The results revealed that the largest group of acetylated proteins was involved in metabolic pathways (20.5%), biosynthesis of antibiotics (13.3%), ribosome (5.8%), purine metabolism (3.3%), carbon metabolism (2%), beta-Lactam resistance (2.0%), and RNA degradation (1.7%) (Figure 3D). In general, these data strongly suggest that the intracellular metabolic pathway may be strictly regulated by lysine acetylation modification, so the acetylated of enzymes in the metabolic pathway is particularly important in response to bile salt stress in *V. alginolyticus*.

### COG function annotation and subcellular localization analysis of acetylated proteins under bile salt stress in *V. alginolyticus*

**Figure 4:**
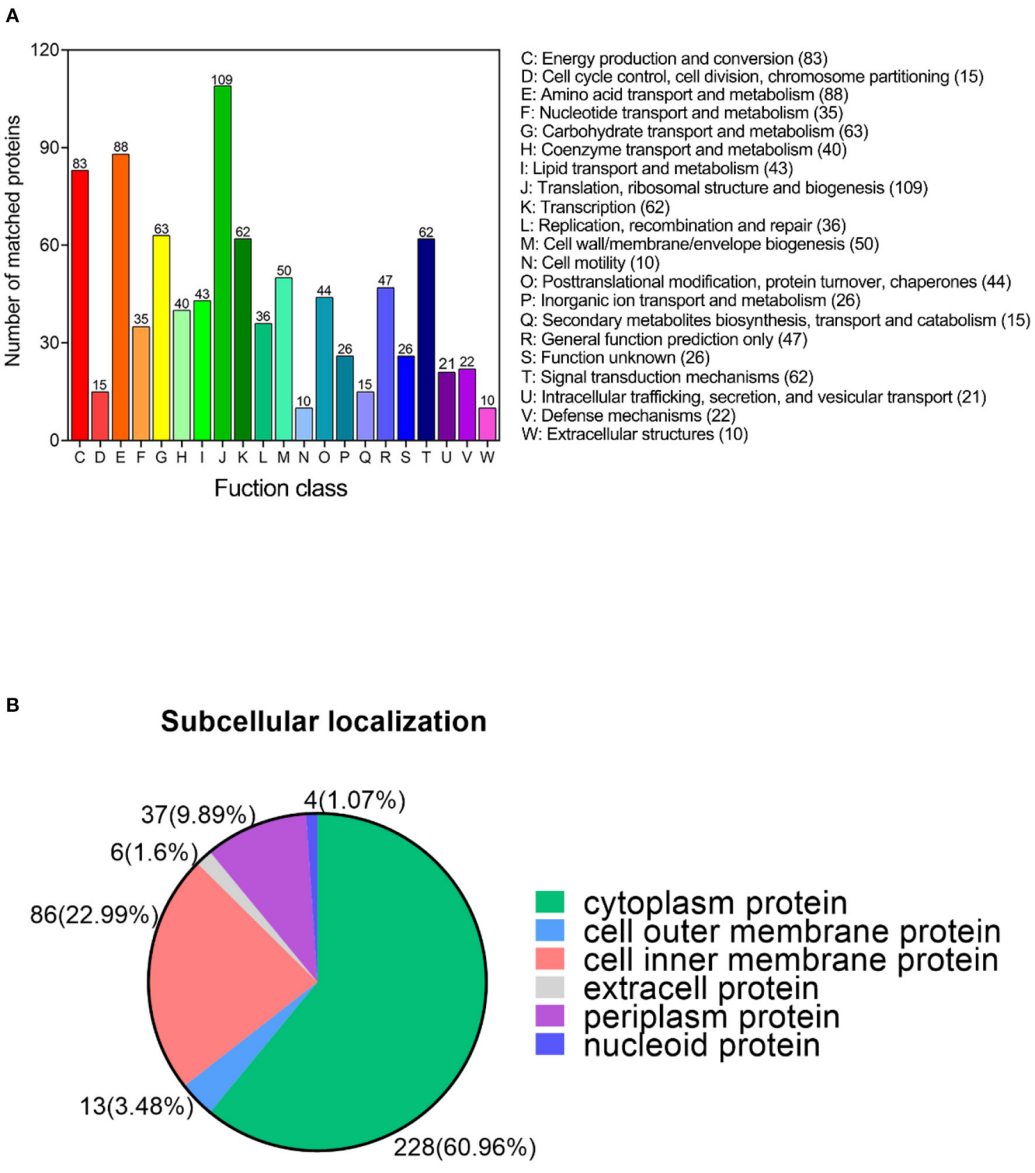
Functional annotation of the lysine acetylated proteins under bile salt stress in *V. alginolyticus*. **(A)** The COG function classification analysis and **(B)** subcellular localization analysis of the acetylated proteins.

Cluster of Orthologous Groups of proteins (COG) functional annotation provides important information proteins function. Annotated acetylated proteins were categorized into 21 functional groups according to the COG function classification. Among these COG categories, a large number of acetylated proteins are enriched in translation, ribosomal structure and biogenesis, amino acid transport and metabolism, energy production and conversion, carbohydrate transport and metabolism, transcription, signal transduction mechanisms, cell wall/membrane/envelope biogenesis, general function prediction only, posttranslational modification, protein turnover, chaperones, and lipid transport and metabolism ten COG terms (Figure 4A). Our results show that a large number of translation and metabolism related proteins undergo acetylation modification.

It is well-known that the biological cell is a highly ordered structure subdivided into multiple membrane-bound compartments with different functions. To explore the distribution of acetylated proteins among cellular compartments, we performed subcellular localization analysis of acetylated proteins using the Cell-mPLOC 2.0 website. Results showed that acetylated proteins were mainly distributed in the cytoplasm (60.96%), followed by the cell inner membrane (22.99%), periplasm (9.98%), cell outer membrane (3.48%), extracell (1.6%), and nucleoid (1.07%) (Figure 4B).

### Virulence factors of acetylated proteins under bile salt stress in *V. alginolyticus*

Through VFDB database, we screened 22 virulence factors in the lysine acetylome of *V. alginolyticus* under bile salt stress (Table 1). These 22 acetylated proteins participate in various pathogenic processes of bacteria through the secretion system (AOG25_14550, *VMC_23950, K05K4_27280, AOG25_21875, secA, secD, secY, tolC, tatA, yajC, yidC*, and *clpB*), chemotaxis and motility (*AOG25_15960, cheA, cheY, frr, AOG25_15815, HUO05_08750*, and *N646_1361*), adherence (*N646_1788* and *VMC_30830*), and toxin (*AOG25_14550*). Acetylation modification of virulence factors may play an important role in the pathogenicity of *V. alginolyticus*.

**Table 1:** Virulence factors in *V. alginolyticus* under bile salt stress.

**Accession**	**Gene**	**Kac sites**	**VF class**
C0LZR3	*AOG25_14550*	K62; K73; K93	Secretion system
D0WYZ5	*VMC_23950*	K46	Secretion system
A0A1W6TUR3	*K05K4_27280*	K120	Secretion system
A0A0P7EMC3	*AOG25_21875*	K61	Secretion system
D0X0B8	*secA*	K539; K729	Secretion system
A0A1W6UHJ5	*secD*	K195; K391	Secretion system
A0A1W6VAU8	*secY*	K348	Secretion system
B3G3J0	*tolC*	K159	Secretion system
A0A1W6VHT2	*tatA*	K48; K53	Secretion system
A0A0G9M1I4	*yajC*	K69	Secretion system
A0A0H0YHX3	*yidC*	K386	Secretion system
A0A0H0Y8A6	*clpB*	K325; K476; K738	Secretion system
A0A0H0YF90	*AOG25_15960*	K20	Chemotaxis and motility
A0A1W6TTD5	*cheA*	K463; K658	Chemotaxis and motility
Q2PED8	*cheY*	K120	Chemotaxis and motility
A0A0G9M2M6	*frr*	K21	Chemotaxis and motility
A0A0G9LVQ9	*AOG25_15815*	K57; K237	Chemotaxis and motility
A0A0S3JVM7	*HUO05_08750*	K145	Chemotaxis and motility
A0A2I3C8B0	*N646_1361*	K9	Chemotaxis and motility
A0A2I3CB94	*N646_1788*	K59; K69; K145	Adherence
A0A1W6V3D0	*VMC_30830*	K469	Adherence
C0LZR3	*AOG25_14550*	K62; K73; K93	Toxin

### Protein-protein interaction networks of the acetylated proteins under bile salt stress in *V. alginolyticus*

**Figure 5:**
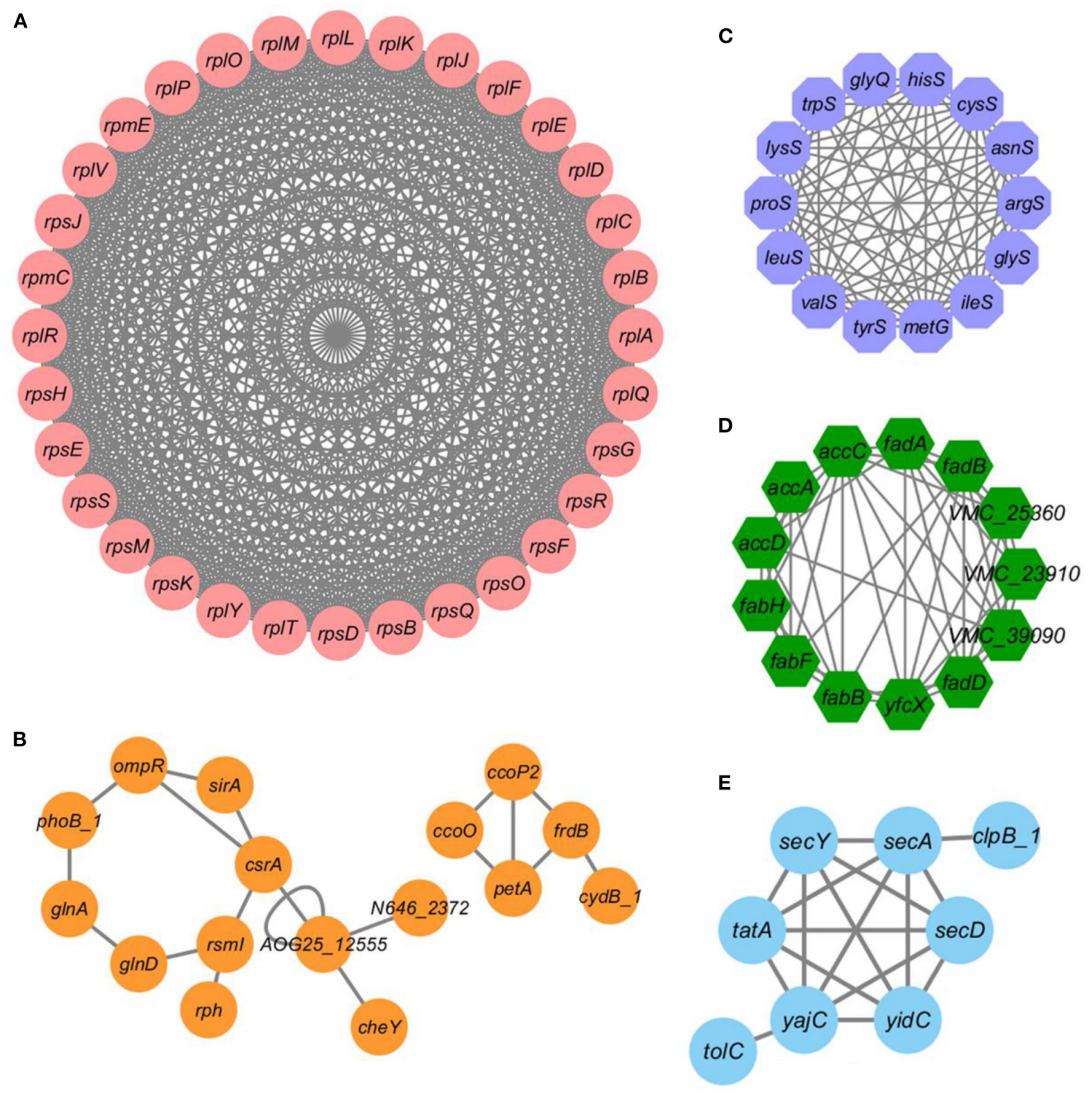
Protein-protein interaction networks of acetylated proteins under bile salt stress in *V. alginolyticus*. PPI networks of acetylated proteins were analyzed using the STRING combine with Cytoscape software. **(A)** ribosomes, **(B)** two-component system, **(C)** aminoacyl-tRNA biosynthesis, **(D)** fatty acid metabolism, and **(E)** bacterial secretion system.

In order to understand the cellular process regulated by lysine acetylation in *V. alginolyticus* under bile salt stress, based on STRING and Cytoscape_ 3.1.7 software generates the protein-protein interaction network. The results showed that 647 acetylated proteins were mapped to the protein network database, forming a highly interconnected protein network. As shown in Figure 5, ribosomes, two-component system, aminoacyl-tRNA biosynthesis, fatty acid metabolism, and bacterial secretion system formed prominent and highly connected protein clusters. In particular, the identified top clusters related to ribosomes, total of 32 network nodes (acetylated proteins) and 496 lines (interactions between proteins) constitute the interaction network (Figure 5A). Other subnetworks for two-component system, fatty acid metabolism, and bacterial secretion system were also have a relatively high density. The complex protein interaction network of lysine acetylated proteins indicates that lysine acetylation modification may regulate a variety of pathways in *V. alginolyticus* under bile salt stress.

### Co-immunoprecipitation and western blotting were used to verify OmpN, OmpR, and GrpE acetylated proteins

**Figure 6:**
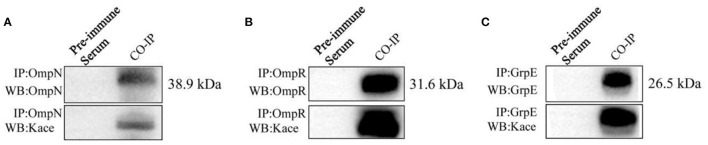
Validation of OmpN **(A)**, OmpR **(B)**, and GrpE **(C)** by Co-IP and Western blotting. OmpN, OmpR, and GrpE proteins were captured by specific antibodies (anti-OmpN, anti-OmpR, and anti-GrpE), and validation by Western blotting with anti-OmpN, anti-OmpR and anti-GrpE (above), and anti-lysine acetylation antibodies (below).

OmpN, OmpR, and GrpE three acetylated proteins, were analyzed by co-immunoprecipitation (Co-IP) and Western blotting to further prove the identified lysine-acetylated results in *V. alginolyticus* under bile salt stress. The OmpN, OmpR, and GrpE proteins were enriched with anti-OmpN, anti-OmpR, and anti-GrpE antibodies and visualized via Western blotting, which was performed with target proteins or anti-acetyllysine antibodies, respectively (Figure 6). The results clearly showed that OmpN, OmpR and GrpE proteins were acetylated.

### Overlap between un-treated and treated with bile salt stress lysine acetylated proteins in *V. alginolyticus*

**Figure 7:**
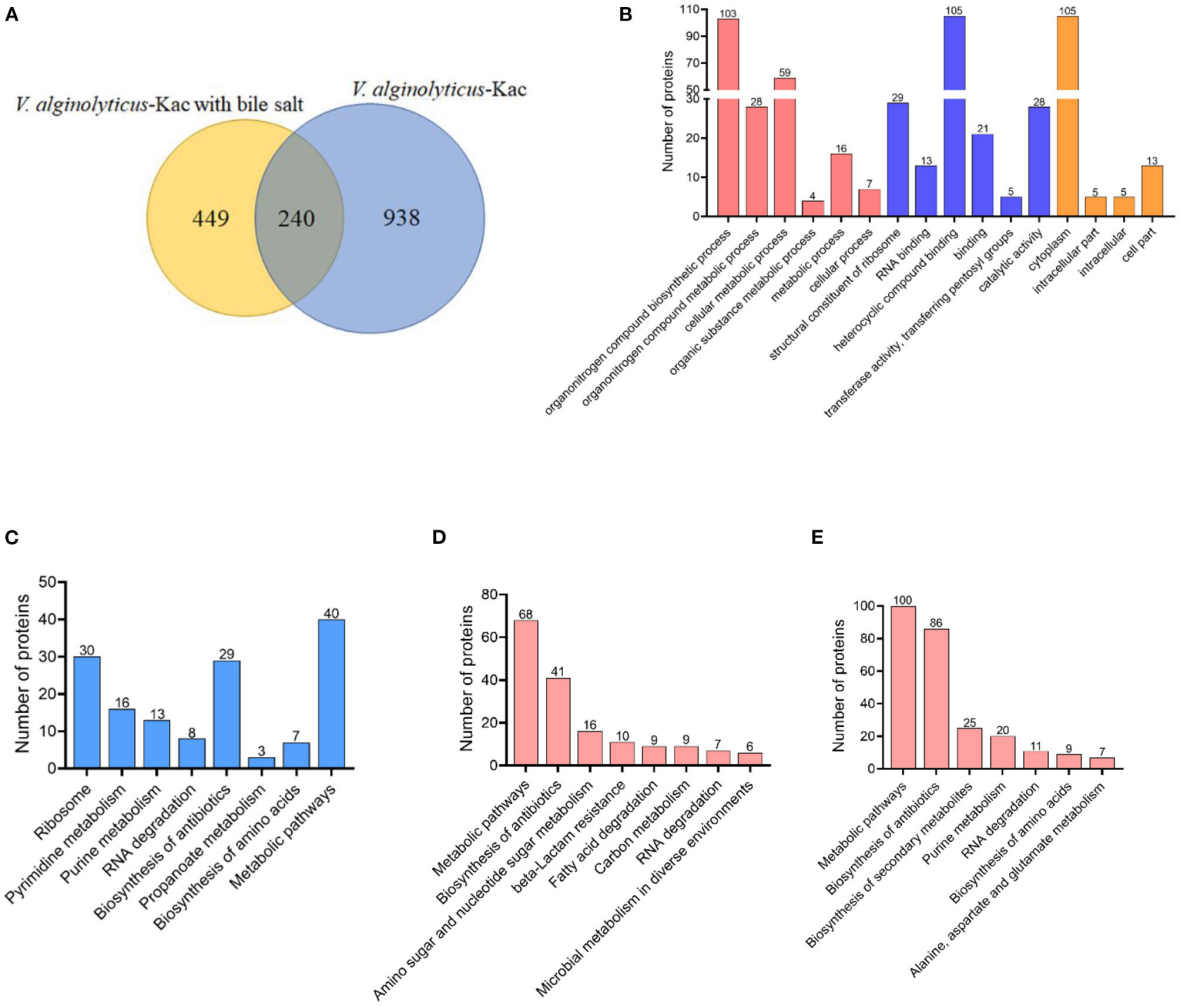
Comparison of un-treated and treated with bile salt stress lysine acetylated proteins in *V. alginolyticus*. **(A)** Overlap between un-treated and treated bile salt stress lysine acetylated proteins in *V. alginolyticus*, **(B)** GO enrichment analysis, and **(C)** KEGG pathway enrichment analysis of the overlapped acetylated proteins. **(D, E)** KEGG pathway enrichment analysis of treated and un-treated with bile salt stress lysine acetylation protein.

According to our previous research report, 1,178 acetylated proteins were identified in *V. alginolyticus* (12), while in this study, we identified 689 proteins that were acetylated under bile salt stress. Venny analysis showed that a total of 240 proteins overlapped (Figure 7A). Go and KEGG analysis were performed on these overlapped proteins. Of the overlapped proteins, GO analysis showed that proteins were enriched into six biological processes, six molecular functions and four cell components classifications. Among them, organonitrogen compound biosynthetic process, heterocyclic compound binding and cytoplasm occupy the dominant position in the biological process, molecular function and cell component classifications (Figure 7B). Further analysis of KEGG pathway showed that most acetylated proteins were involved in the regulation of metabolic pathways, ribosome, biosynthesis of antibiotics, pyrimidine metabolism and purine metabolism (Figure 7C). KEGG analysis of treated and un-treated with bile salt stress lysine acetylation protein (449 and 938 proteins) of *V. alginolyticus* showed that it was enriched into 8 and 7 metabolic pathways, respectively (Figures 7D, E). The same pathways are metabolic pathways, biosynthesis of antibiotics and RNA degradation. Under bile salt stress amino sugar and nucleotide sugar metabolism, beta-Lactam resistance, fatty acid degradation, carbon metabolism, and microbial metabolism in diverse environments were significantly enriched (Figure 7D). However, biosynthesis of secondary metabolites, purine metabolism, biosynthesis of amino acids, and alanine, aspartate and glutamate metabolism were significantly enriched in the absence of bile salt stress (Figure 7E).

## Discussion

Acetylation is an important post-translational modification in bacteria, which is necessary to maintain cellular homeostasis, regulate transcription and translation of genes, and alter cellular metabolism based on environmental stress responses (1, 35). Bile salts can trigger the expression of bacterial virulence factors, leading to changes in various intracellular biological processes in cell (23). However, up to now, there is limited information about the role of acetylation of *V. alginolyticus* involved in the regulation of under bile salts stress and its regulatory mechanisms are still unclear. In this study, 1,315 unique lysine-acetylated peptides on 689 proteins [account for 15.88% (689/4,338) of the total proteins in *V. alginolyticus*] were identified under bile salt stress in *V. alginolyticus*. This ratio was higher than that of the aquatic pathogens *V. mimicus* (15.5%) and *V. parahaemolyticus* (13.6%), but lower than that of *V. alginolyticus* (27.1%), *A. hydrophila* (21.7%), and *V. vulnificus* (40.34%) (9–12, 36).

Many previous studies reported that chaperones changed significantly under bile salt stress. In this study, 13 chaperones were also found to be acetylated, including DanK, TorD, ClpX, YidC, Tig, HslU, GrpE, ClpA, ClpB, RimM, Hsp60, GroEL, and GroL-2. These chaperones play a key role in the accurate integrity and stability of intracellular biological molecules (such as protein and DNA) and resistance to bile salt stress (37–40). Furthermore, FrdA, FrdB, SdhB, SucB, SucC, SucD, Mdh, GltA, AceB, PdhA, AcnB, and Did 12 key enzymes of TCA cycle also undergo acetylation modification. The results show that acetylated chaperones and metabolic processes related proteins play an important role in the corresponding bile salt stress and reducing damage and energy efficiency in *V. alginolyticus*.

The identified acetylated proteins were then further examined using GO, KEGG pathway, COG function classification and subcellular localization analyses, and the result showed that many of the acetylated proteins contributed to complicated cellular metabolic process. For example, under bile salt stress environment, translation, ribosomal structure and biogenesis, amino acid transport and metabolism, and energy production and conversion were enriched in *V. alginolyticus*. Furthermore, ribosome and purine metabolism related proteins are higher tendency to be acetylated, and this result have also been reported in *V. vulnificus, M. tuberculosis* and *V. parahaemolyticus* (11, 36, 41). As well as the acetylated modified proteins are mainly distributed in the cytoplasm and are highly conserved, which is similar to the results previously obtained in *Spiroplasma eriocheiris, Brenneria nigrifluens, Streptococcus pneumoniae*, and *Streptococcus mutans* (42–45). These findings suggest that the noted proteins involved in translation, protein synthesis and energy metabolism tend to undergo acetylation modification in cytoplasm under bile salt stress.

Interesting, under bile salt stress sample, many of proteins as virulence factors were shown to be acetylated. For example, secretion system related genes *secA, secD*, secF, *yajC, yidC* and *tolC*, which prior to studies reported that secretion system genes played roles in the bacterial adhesion, thereby promoting the adhesion of *V. alginolyticus* to the host surface and causing bacterial infection (46). In *Vibrio cholerae*, the outer membrane protein TolC is necessary for the expression of *toxR* regulon and participates in an efflux dependent feedback loop to regulate the expression of virulence genes, MARTX toxin secretion and resistance to antimicrobial compounds present in the host (47). In this study, we also found that ToxR protein, a key transcription activator, is acetylated. Bile salt can promote the activation of *toxR* regulator and participate in the regulation of bacterial virulence genes (48, 49). Bacterial toxin factor (*AOG25_14550*) is a Hcp1 family type VI secretion system effector, which also acetylated under bile salt stress in *V. alginolyticus*. It is reported that it is related to bacterial virulence in *Edwardsiella ictaluri* and *A. hydrophila* (50, 51). Furthermore, in this study, many chemotaxis, motility and adherence related proteins are acetylated. A large number of studies have proved that bacteria can move to the favorable environment or away from the harmful environment through chemotaxis and motility, which plays an important role in infection and disease. Therefore, it can block infection and prevent disease by interfering with chemotaxis and motor signaling pathways (52, 53). Additionally, Type IV pilus, mannose-sensitive hemagglutinin A (*N646_1788*) and MSHA biogenesis protein MshH (*VMC_30830*) participate in the regulation of bacterial surface attachment and colonization by controlling the dynamic activity of pili, especially in *Vibrio cholerae* (54, 55). In conclusion, a variety of virulence factors in *V. alginolyticus* are acetylated under bile salt stress, and jointly participate in the regulation of bacterial pathogenicity.

Additionally, protein-protein interaction network plays an important role in regulating cells and their signals. In this study, the ribosome related protein network is the most complex, which is consistent with many previous research results. Advanced complex composed of ribosome related proteins was found in many prokaryotic bacteria, such as *V. parahaemolyticus* (45 acetylated proteins and 982 direct physical interactions), *V. mimicus* (36 acetylated proteins and 630 direct physical interactions), *Shewanella baltica* (43 acetylated proteins), and *Streptomyces coelicolor* (25 acetylated proteins) (10, 11, 56, 57) are mainly have a hand in protein translation. Other subnetworks of proteins related to two-component system, fatty acid metabolism, and bacterial secretion system also have a relatively high correlation, and these pathways are considered to be closely related to bacterial pathogenicity (58–60). The complex protein interaction network of lysine acetylated proteins indicates that lysine acetylation modification may regulate a variety of pathways in *V. alginolyticus* under bile salt stress and participate in the regulation of bacterial virulence. Protein-protein interaction network provides a better approach for understanding the functions of acetylated proteins in cells. In a word, this study reported the global acetylation proteome of *V. alginolyticus* under bile salt stress, which provided a basis for the future study of acetylation regulation function and molecular mechanism of *V. alginolyticus* under bile salt stress.

## Data availability statement

The datasets presented in this study can be found in online repositories. The names of the repository/repositories and accession number(s) can be found in the article/Supplementary material.

## Ethics statement

All animal experiments were conducted strictly based on the recommendations in the Guide for the Care and Use of Laboratory Animals set by the National Institutes of Health. The animal protocols were approved by the Animal Ethics Committee of Guangdong Ocean University (Zhanjiang, China). In this study, there was no interaction with *Lutjanus erythopterus* from which the *Vibrio alginolyticus* HY9901 was isolated during the previous study. The bacteria protocols were approved by the Biosecurity Committee of Guangdong Ocean University (Zhanjiang, China).

## Author contributions

HP designed the study and wrote the manuscript. WL analyzed the data and wrote the manuscript. XX, YP, ZW, and SW generated experimental data. JW wrote the manuscript. NW and JJ critically reviewed the manuscript. All authors contributed to the article and approved the final version of the manuscript.

## Supplementary material

Supplementary Table 1 Acetylated proteins and sites identified under bile salt stress in *V. alginolyticus*.

Supplementary Table 2 The identified peptides under bile salt stress in *V. alginolyticus*.
