# Supplementary material for: Holistic analysis of lysine acetylation in aquaculture pathogenic bacteria Vibrio alginolyticus under bile salt stress
Source: Front Vet Sci. 2023 Apr 27;10:1099255. doi: 10.3389/fvets.2023.1099255 (PMC10172577; doi:10.3389/fvets.2023.1099255)

**Supplement Figure**

Validation of OmpN, OmpR and GrpE by Co-Immunoprecipitation and Western blotting.


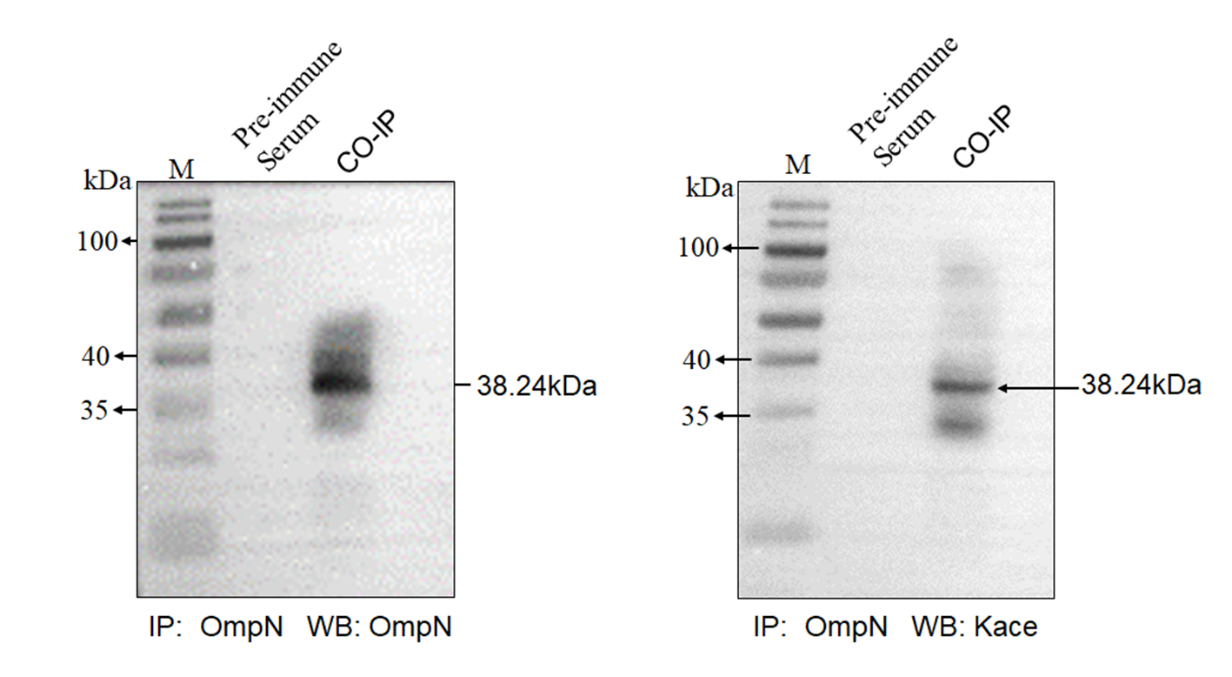


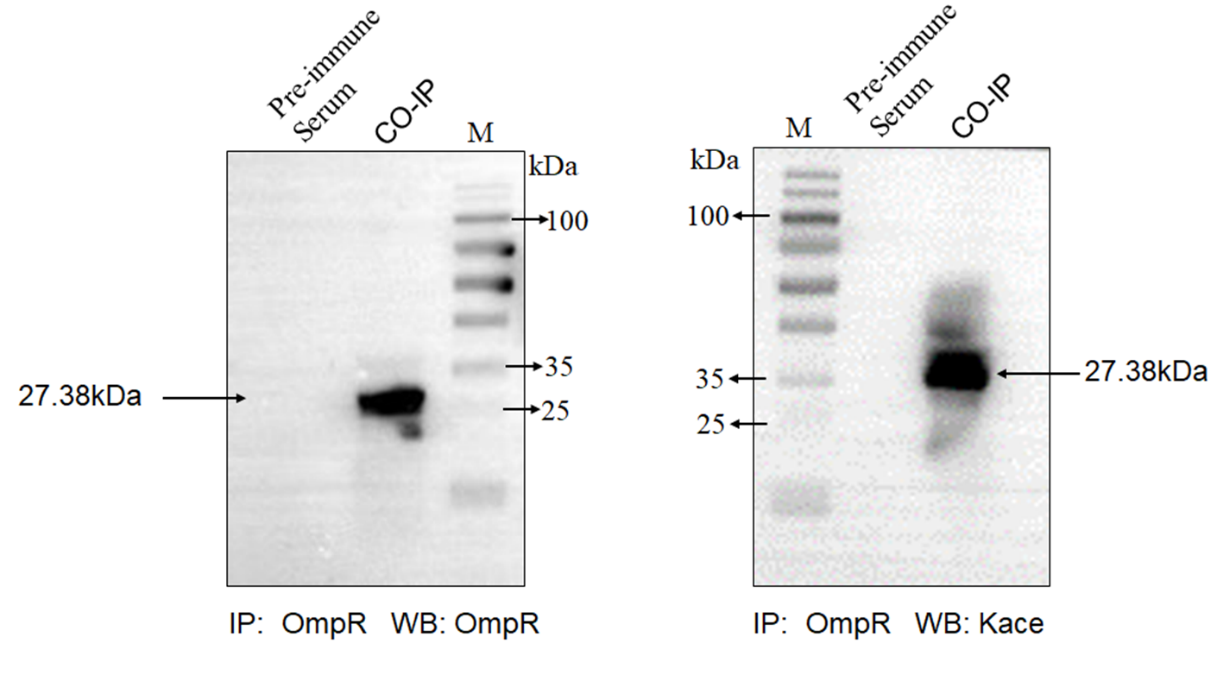


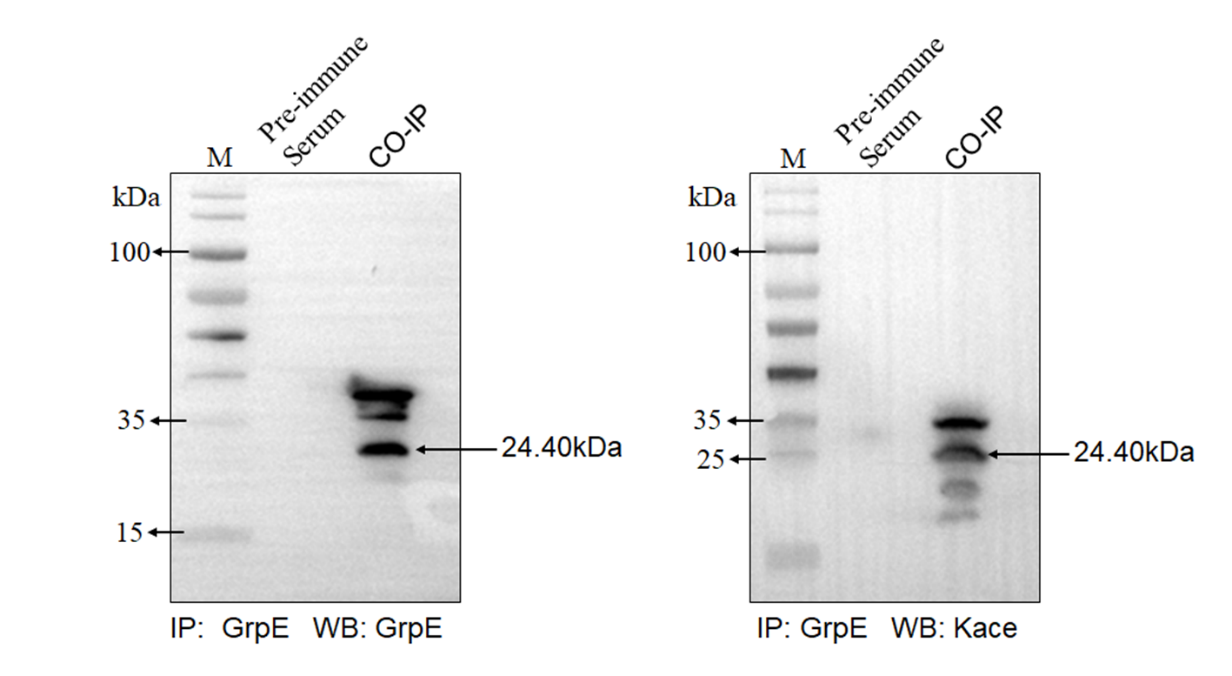

Supplement: Supplementary file 3 [file Data_Sheet_1.docx]
